# Poster Session II – Poster of Distinction II - A315 THE IMPACT OF SOCIOECONOMIC AND DEMOGRAPHIC FACTORS ON TREATMENT OUTCOMES IN PATIENTS WITH PANCREATIC FLUID COLLECTIONS

**DOI:** 10.1093/jcag/gwaf042.315

**Published:** 2026-02-13

**Authors:** G S Brar, M Mahjoob, K Khalaf, K Pawlak, T He, Y Fujiyoshi, M A Bucheeri, H Li, R Gholami, J Mosko, C W Teshima, G May, N Calo

**Affiliations:** Gastroenterology, University of Toronto Temerty Faculty of Medicine, Toronto, ON, Canada; Gastroenterology, University of Toronto Temerty Faculty of Medicine, Toronto, ON, Canada; Medicine, Div of Gastroenterology, St Michael’s Hospital, Toronto, ON, Canada; Medicine, Div of Gastroenterology, St Michael’s Hospital, Toronto, ON, Canada; Medicine, Div of Gastroenterology, St Michael’s Hospital, Toronto, ON, Canada; Medicine, Div of Gastroenterology, St Michael’s Hospital, Toronto, ON, Canada; Medicine, Div of Gastroenterology, St Michael’s Hospital, Toronto, ON, Canada; Gastroenterology, University of Toronto Temerty Faculty of Medicine, Toronto, ON, Canada; Medicine, Div of Gastroenterology, St Michael’s Hospital, Toronto, ON, Canada; Medicine, Div of Gastroenterology, St Michael’s Hospital, Toronto, ON, Canada; Medicine, Div of Gastroenterology, St Michael’s Hospital, Toronto, ON, Canada; Medicine, Div of Gastroenterology, St Michael’s Hospital, Toronto, ON, Canada; Medicine, Div of Gastroenterology, St Michael’s Hospital, Toronto, ON, Canada

## Abstract

**Background:**

Pancreatic fluid collections (PFCs) cause significant morbidity and healthcare burden. While minimally invasive drainage improves outcomes, the influence of social determinants of health (SDOH) on access, treatment, and outcomes remains underexplored.

**Aims:**

We evaluated associations between SDOH and long-term outcomes in patients undergoing PFC drainage at a tertiary care center.

**Methods:**

We conducted a retrospective cohort study of consecutive adults who underwent PFC drainage at St. Michael’s Hospital, Toronto, from January 2016 to February 2024. Patient postal codes were linked to 2021 Canadian Census data to estimate income, housing, education, language, and Indigenous identity. Outcomes included clinical improvement (resolution of drainage indication), radiologic resolution, and hospital readmission. Bivariate and multivariable logistic regression assessed associations.

**Results:**

Among 193 patients (median age 53.9 years, 63.7% male), 91.1% underwent initial endoscopic drainage, most often for infection (27.0%). Bivariate analysis revealed that Indigenous identity was associated with greater use of percutaneous drainage (p = 0.026), and higher rates of abdominal pain (p = 0.010), opioid (p = 0.001), and antibiotic prescriptions (p = 0.017) at discharge. Substance use disorder (SUD) was linked to alcohol-related pancreatitis (p < 0.001), pain at discharge (p = 0.022), prolonged PFC duration (p = 0.005), and higher readmission rates (p = 0.012). Lower median income was associated with reduced rates of additional endoscopic drainage (p = 0.043).

In multivariable models, Indigenous identity (OR 0.864 [0.779–0.958], p = 0.005), shelter cost (OR 0.251 [0.093–0.671], p = 0.006), and post-secondary education (OR 0.938 [0.892–0.987], p = 0.014) were associated with lower odds of clinical improvement. Higher income (OR 1.100 [1.019–1.189], p = 0.015) and spending >30% of income on shelter (OR 1.124 [1.057–1.195], p < 0.001) predicted greater improvement. SUD was linked to reduced odds of radiologic resolution (OR 0.436 [0.183–0.999], p = 0.049). The income-to-shelter cost ratio predicted readmission (OR 1.031 [1.004–1.060], p = 0.028).

**Conclusions:**

SDOH, including Indigenous identity, income, housing burden, and SUD, were associated with disparities in treatment and outcomes following PFC drainage. These findings support integrating social risk screening and equity-focused care pathways to improve outcomes for vulnerable populations.

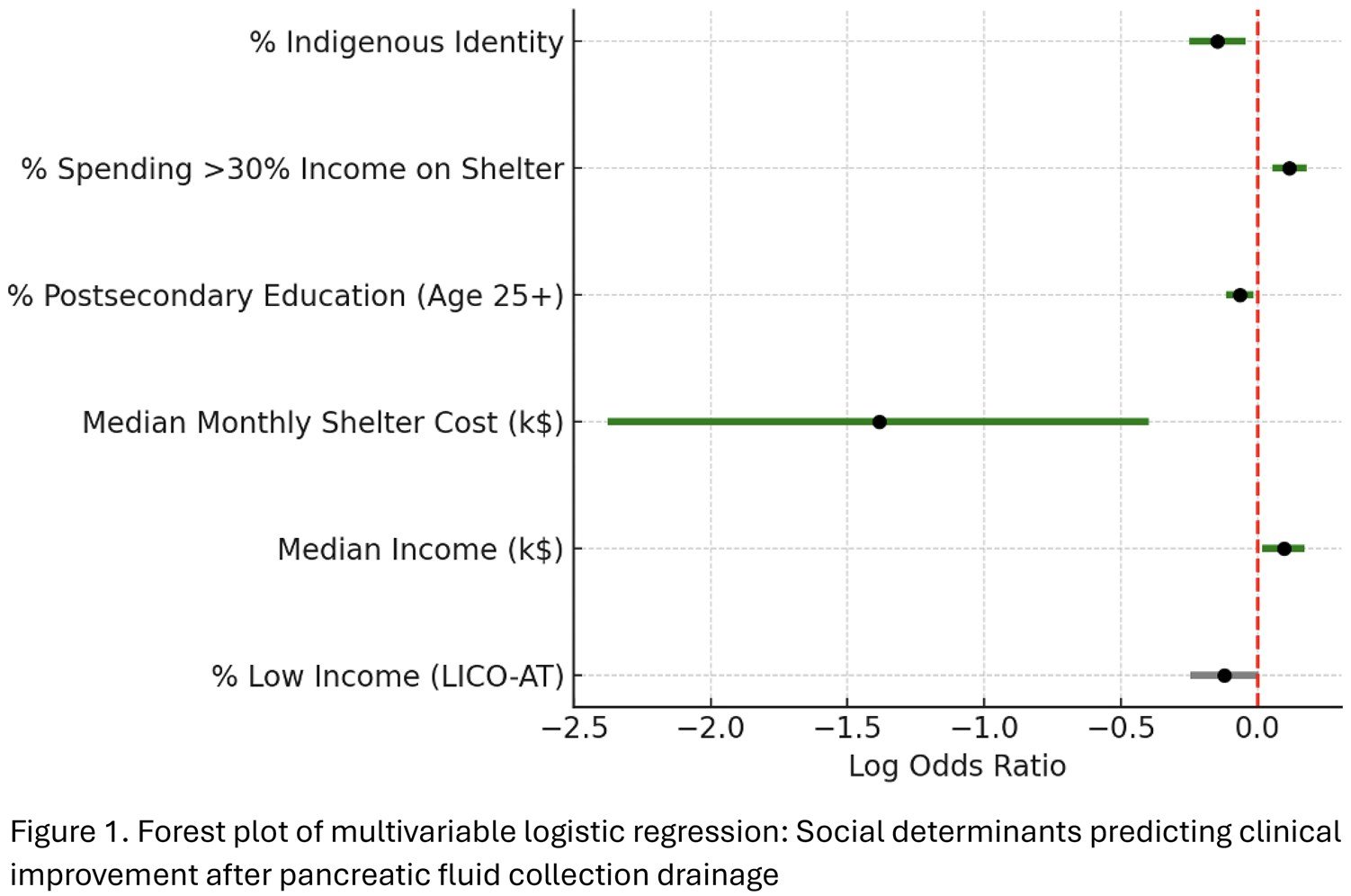

**Funding Agencies:**

None

